# The association between autonomic dysfunction, inflammation and atherosclerosis in men under investigation for carotid plaques

**DOI:** 10.1371/journal.pone.0174974

**Published:** 2017-04-04

**Authors:** Marcus A. Ulleryd, Ulrica Prahl, Johannes Börsbo, Caroline Schmidt, Staffan Nilsson, Göran Bergström, Maria E. Johansson

**Affiliations:** 1 Department of Physiology, Institute of Neuroscience and Physiology, The Sahlgrenska Academy, University of Gothenburg, Gothenburg, Sweden; 2 Department of Molecular and Clinical Medicine, Wallenberg Laboratory for Cardiovascular and Metabolic Research, University of Gothenburg, Sahlgrenska University Hospital, Gothenburg, Sweden; 3 Department of Mathematical Statistics, Chalmers University of Technology, Gothenburg, Sweden; Ludwig-Maximilians-Universitat Munchen, GERMANY

## Abstract

**Background:**

Autonomic dysfunction is a risk factor for cardiovascular disease (CVD), however, the exact mechanism linking autonomic dysfunction to cardiovascular disease is not known. In this study we hypothesized that autonomic dysfunction increases inflammation, which subsequently accelerates atherosclerosis. The aim of the current study was to investigate the association between autonomic tone, inflammation and atherosclerosis.

**Methods:**

124 men under investigation for carotid atherosclerosis were examined for autonomic function (heart rate variability; HRV and baroreflex sensitivity; BRS), inflammatory markers (white blood cell count; WBCC and C-reactive protein; CRP) and degree of carotid atherosclerosis. The direct or indirect associations between autonomic function, inflammatory parameters and carotid plaque area were investigated with multiple linear regressions.

**Results:**

Male subjects with prevalent CVD showed larger carotid plaque area, higher WBCC, and reduced BRS compared to subjects with no history of CVD. Further, BRS was inversely associated with carotid plaque area (r = -0.21, p = 0.018) as well as inflammatory parameters WBCC and CRP (r = -0.29, p = 0.001, and r = -0.23, p = 0.009, respectively), whereas HRV only was inversely associated with WBCC (r = -0.22, p = 0.014). To investigate if inflammation could provide a link between autonomic function and carotid atherosclerosis we adjusted the associations accordingly. After adjusting for WBCC and CRP the inverse association between BRS and carotid plaque area was attenuated and did not remain significant, while both WBCC and CRP remained significantly associated with carotid plaque area, indicating that low-grade inflammation can possibly link BRS to atherosclerosis. Also, after adjusting for age, antihypertensive treatment and cardiovascular risk factors, BRS was independently inversely associated with both WBCC and CRP, and HRV independently inversely associated with WBCC. WBCC was the only inflammatory marker independently associated with carotid plaque area after adjustment.

**Conclusions:**

We demonstrate that autonomic dysfunction is associated with atherosclerosis and that inflammation could play an important role in mediating this relationship.

## Introduction

Autonomic dysfunction is associated with increased risk of cardiovascular disease (CVD) and mortality [1, 2]. Autonomic dysfunction has also been associated with increased atherosclerosis, the main underlying cause of CVD [3]. The mechanisms linking the autonomic imbalance to atherosclerosis are still elusive. Recent studies suggest an association between autonomic function and inflammation in patients with CVD, showing an inverse relationship between autonomic activity, measured by heart rate variability (HRV), and plasma levels of inflammatory markers [4–7]. HRV is a well-established and widely used approach to measure autonomic function in humans and represents the time differences between successive heartbeats [8]. However, other measures of autonomic tone like baroreflex sensitivity, assessed by analyzing the relationship between fluctuations in blood pressure and corresponding changes in RR intervals, have been poorly assessed for inflammatory associations and needs to be further investigated.

Inflammation is a key mediator in the pathophysiology of atherosclerosis involving both the innate and the adaptive immune system [9, 10]. Further, inflammatory markers such as white blood cell count (WBCC) and C-reactive protein (CRP) can predict CVD [11–13], and have been associated with atherosclerosis [14–20]. In line with this, the prevalence of CVD is known to be higher in subjects with autoimmune disorders, like systemic lupus erythematosus and rheumatoid arthritis [21, 22]. Given this background we hypothesize that inflammation can be a mediator in the link between autonomic dysfunction and atherosclerosis, leading to CVD like stroke and myocardial infarction. Few studies have investigated the whole pathway (autonomic tone—inflammation—atherosclerosis) in the same population, or measured carotid atherosclerosis as the primary end-point.

We hypothesized that reduced autonomic function will increase low-grade inflammation, subsequently worsening the progression of atherosclerosis. The aim of the current study was therefor to investigate if there is a relationship between autonomic function, assessed by both HRV and BRS, the inflammatory markers CRP and WBCC, and carotid plaque area in men undergoing investigation for carotid plaques. Further, we aimed to investigate if inflammation may be a linking mechanism between autonomic dysfunction and atherosclerosis.

## Materials and methods

### Study population

The participants were consecutively recruited from the “The Western Region Initiative to Gather Information on Atherosclerosis” (WINGA) population on the basis that they were males and ≥ 40 years old. The WINGA population provides patient records of all patients undergoing clinical diagnostic carotid ultrasound examinations within the Greater Gothenburg region in Sweden due to any symptoms, *i*.*e*. sudden paralysis, numbness, loss of speech, loss of vision, dizziness or severe and unusual headache. Inclusion criteria were successful assessment of autonomic function, plasma levels of C-reactive protein (CRP) ≤ 10 mg/L and white blood cell count (WBCC) ≤ 30 cells 10^9^/L. Subjects with rheumatoid disease were excluded. Applying above mentioned criteria, a total of 124 subjects were enrolled. The study protocol conforms to the ethical guidelines of the 1975 Declaration of Helsinki and approval was obtained from the Regional Ethics Committee in Gothenburg. All patients gave written informed consent to participate.

### Baseline characteristics

Subjects were invited to the laboratory and detailed clinical and life style data were collected using a standardized questionnaire. Data on anthropometry and blood pressure were collected and venous blood samples were drawn. Inflammatory status was evaluated by measuring WBCC and high-sensitivity CRP.

### Measurement of carotid plaque area

Plaque area was assessed by 2D ultrasound in the common carotid artery (CCA), the carotid bulb and the proximal section of the internal carotid artery (ICA), bilaterally, as previously described [23]. Plaques were defined according to the Mannheim consensus [24]. Images were obtained of each atherosclerotic plaque and they were semi-automatically outlined and measured using a dedicated software [23]. Re-reading reproducibility of plaque size (n = 45) showed high correlation coefficients for the right and left carotid arteries (rs = 0.96 and rs = 0.96, respectively)[25].

### Assessment of autonomic function

Autonomic function was assessed by measuring heart rate variability and baroreflex sensitivity in subjects.

#### Heart rate variability (SDNN)

A 3-lead electrocardiographic (ECG) recording was performed in supine position for 20 minutes with Biopac Pro 3.7 software (BIOPAC Systems Inc, Goleta, CA, USA). Time domain analysis on the standard deviation (SD) between normal to normal RR -intervals in the ECG-recording (SDNN) was derived with the Baro Reflex Analysis (BRA) software (Ekman Biomedical Data AB, Gothenburg, Sweden). SDNN reflects both sympathetic and parasympathetic variability during the time of recording [8].

#### Baroreflex sensitivity (Slope)

Continuous recordings of blood pressure were made with the sequence method [26] using The CNAP^**™**^ Monitor 500 (CNSystems Medizintechnik AG, Graz, Austria) and Biopac PRO 3.7 software (BIOPAC Systems Inc) over a period of 20 minutes with the ECG recording simultaneously. A baroreflex sequence was defined as 3 consecutive beats when the RR interval follows an increase or decrease in the systolic blood pressure, with the threshold set at 5 ms and 1 mmHg, respectively. A linear regression was applied to sequences, and correlation coefficients with r >0.92 were accepted. A mean slope was calculated from the average of qualified sequences.

A limit of 5 percent data loss in both HRV and BRS analysis was used as exclusion criteria. These kinds of data losses could be technical or biological, such as cardiac arrhythmias.

### Statistics

Characteristics between different patient categories were compared using Student´s t-test for continuous variables and Chi-square test for categorical variables. Normal distribution was evaluated using Kolmogorov-Smirnov test. BRS Slope, HRV SDNN, CRP, WBCC, carotid plaque area, total cholesterol, LDL-cholesterol and HDL-cholesterol were logarithmically transformed due to skewness in distribution. However, untransformed data was presented as mean ± SD for better comparison with data from other studies.

The association between parameters for autonomic tone (HRV SDNN and BRS Slope), traditional risk factors for atherosclerosis, inflammatory markers, medical therapy and carotid plaque area were assessed with correlation (Pearson) analyses. To assess if inflammation could mediate the association between autonomic function (BRS and HRV) and carotid plaque area, multiple linear regression models with 2 predictors were used, adjusting for CRP and WBCC in separate analysis.

Independent associations between parameters for autonomic tone and inflammation, and between parameters for inflammation and atherosclerosis were assessed with multiple linear regression, adjusting for medical therapy and clinical factors. Model 1 adjusted for age. Model 2 adjusted for age and antihypertensive medicine. Model 3 was a stepwise adjustment for prevalent CVD, current smoker, BMI, diabetes, hypertension, dyslipidemia, systolic blood pressure and cholesterol-lowering medicine with a selection of p<0.05 for inclusion. Age and antihypertensive medicine were forced into the model.

The analyses were additionally stratified for treatment with statins and differences in regression slopes evaluated with an interaction term between statins and the main predictor in the model.

All data was analyzed with SPSS (IBM SPSS Statistics version 24.0, Chicago, IL, USA) and *p*<0.05 was considered as the level of significance.

## Results

### Characteristics of the study population

Analysis was carried out in 124 male subjects undergoing clinical diagnostic carotid ultrasound examinations due to any symptoms. 50% of the study subjects had prevalent CVD, defined as stroke, myocardial infarction or both. The prevalence of subjects ever diagnosed with hypertension, dyslipidemia or diabetes was 68%, 64% and 15%, respectively. The mean age was 67 years. Subject characteristics are listed in Table 1.

**Table 1:** Characteristics of study population and comparison between subjects with or without prevalent CVD.

Variables	Values			
	All subjects (n = 124)	No history of CVD (n = 62)	Prevalent CVD (n = 62)	p*
Smoking, Yes/No	14/110	4/58	10/52	0.09
Diabetes^a^, Yes/No	18/106	4/58	14/48	0.011
Dyslipidemia^a^, Yes/No	77/44	37/24	40/20	0.7
Hypertension^a^, Yes/No	84/39	38/24	46/15	0.1
Cholesterol-lowering medicine Yes/No	64/54	31/29	36/25	0.4
Antihypertensive medicine Yes/No	72/50	31/30	41/20	0.066
Age, years	67.3 ± 8	67 ± 9	68 ± 8	0.7
Carotid plaque area, mm^2^	58.3 ± 51.6	44.1 ± 40.4	73.0 ± 57.9	<0.001
TC, mg/dL	4.6 ± 1.0	5.0 ± 1.1	4.2 ± 0.8	<0.001
LDL, mg/dL	2.7 ± 0.9	3.0 ± 1.0	2.4 ± 0.7	<0.001
HDL, mg/dL	1.4 ± 0.4	1.5 ± 1.1	1.4 ± 0.4	0.060
SBP, mm Hg	135 ± 16	134 ± 16	137 ± 16	0.4
DBP, mm Hg	77 ± 9	78 ± 8	77 ± 9	0.5
BMI, kg/m^2^	26.8 ± 3.7	26.0 ± 3.2	27.5 ± 3.9	0.017
CRP, mg/L	1.81 ± 1.7	1.72 ± 1.61	1.91 ± 1.77	0.3
WBCC, cells 10^9^/L	6.1 ± 1.6	5.8 ± 1.6	6.4 ± 1.7	0.040
HRV SDNN, ms	44.3 ± 18.4	45.9 ± 17.9	42.7 ± 18.9	0.3
BRS Slope, ms/mm Hg	11.1 ± 6.8	12.8 ± 7.9	9.5 ± 5.1	0.022

Values expressed as counts for categorical variables and mean ± standard deviation (SD) for numerical variables. MI: Myocardial infarction, TC: Total cholesterol, LDL: Low-density lipoprotein, HDL: High-density lipoprotein, SBP; Systolic blood pressure, DBP: Diastolic blood pressure, BMI: Body mass index, HR: Heart rate, CRP: C-reactive protein, WBCC: White blood cell count, BRS: Baroreflex sensitivity, HRV: Heart rate variability, SDNN: standard deviation of RR interval.

^a^ Subjects ever diagnosed with the disorder

*p-values for the difference between patients with or without prevalent CVD. BRS Slope, HRV SDNN, CRP, WBCC, carotid plaque area, total cholesterol, LDL-cholesterol and HDL-cholesterol were logarithmically transformed in the analysis. However, untransformed data was presented as mean ± SD for all variables for better comparison with data from other studies.

### Comparison between subjects with prevalent CVD and subjects with no history of CVD

The study population was initially analyzed by comparing variables of inflammation, autonomic function and atherosclerosis in subjects with or without prevalent CVD (n = 62, respectively). Subjects with prevalent CVD showed decreased autonomic function, assessed with BRS Slope, increased WBCC and larger carotid plaque area, compared to subjects with no history of CVD (Table 1). However, there was no difference in plasma levels of the inflammatory marker CRP, or autonomic function assessed by HRV SDNN (Table 1). These data prompted us to expand the investigation on this interrelationship.

### Relationships between autonomic function, inflammation, carotid plaque area and clinical factors

To investigate possible variables with association to carotid plaque area and inflammatory markers, univariate correlations were calculated between corresponding parameters and different clinical factors of interest (Table 2). Carotid plaque area showed a positive correlation with WBCC, CRP, age, prevalent CVD, and antihypertensive medicine whereas BRS Slope was inversely correlated with carotid plaque area. Investigating predictors of inflammation, CRP was inversely correlated with BRS Slope and positively correlated with BMI. Further, WBCC was inversely correlated with BRS Slope and HRV SDNN, and positively correlated with age, smoking, BMI, prevalent CVD and antihypertensive medicine.

**Table 2:** Relationships between parameters for autonomic function, inflammatory markers, atherosclerosis and clinical factors.

Variables	Log carotid plaque area		Log WBCC		Log CRP	
Univariate	r	p	r	p	r	p
CVD Risk Factors						
Age, years	0.31	<0.001	0.21	0.019	0.11	0.2
Smoking	0.14	0.1	0.24	0.007	0,00	1.0
BMI, kg/m^2^	0.14	0.1	0.19	0.030	0.19	0.034
SBP, mm Hg	0.16	0.086	0.15	0.091	0.07	0.5
DBP, mm Hg	-0.08	0.4	-0.11	0.2	-0.03	0.7
Log TC, mg/dL	-0.08	0.4	-0.11	0.2	0.02	0.8
Log LDL, mg/dL	-0.06	0.5	-0.15	0.085	0.03	0.7
Log HDL, mg/dL	-0.11	0.2	-0.16	0.1	<0.00	1.0
Prevalent stroke/MI	0.30	<0.001	0.19	0.040	0.09	0.3
Diabetes^a^	0.08	0.4	0.11	0.2	0.10	0.3
Dyslipidemia^a^	-0.14	0.1	0.014	0.9	-0.14	0.1
Hypertension^a^	-0.01	0.9	-0.04	0.6	-0.15	0.1
Medication						
Cholesterol-lowering	0.07	0.5	0.1	0.2	0.07	0.4
Antihypertensive	0.30	<0.001	0.18	0.040	0.08	0.4
Autonomic function						
Log BRS Slope, ms/mm Hg	-0.21	0.018	-0.29	0.001	-0.23	0.009
Log HRV SDNN, ms	-0.16	0.081	-0.22	0.014	-0.13	0.2
Inflammation						
Log WBCC, mg/L	0.36	<0.001	-	-	0.27	0.003
Log CRP, mg/L	0.22	0.014	0.27	0.003	-	-
2-predictor models	β	p				
Log BRS Slope, ms/mm Hg	-0.11	0.2				
Log WBCC, mg/L	0.32	0.001				
Log BRS Slope, ms/mm Hg	-0.17	0.063				
Log CRP, mg/L	0.18	0.047				
Log HRV SDNN, ms	-0.08	0.4				
Log WBCC, mg/L	0.336	<0.001				
Log HRV SDNN, ms	-0.13	0.1				
Log CRP, mg/L	0.206	0.023				

r-values in univariate analysis are Pearson correlation coefficients and β-values in 2-predictor models are standardized regression coefficients. 2-predictor models are multiple linear regressions including one variable of autonomic function and one variable of inflammation. CRP: C-reactive protein, WBCC: White blood cell count, HRV: Heart rate variability, SDNN: standard deviation of RR interval, BRS: Baroreflex sensitivity, CVD: Cardiovascular disease, TC: Total cholesterol, LDL: Low-density lipoprotein, HDL: High-density lipoprotein, SBP; Systolic blood pressure, DBP: Diastolic blood pressure, BMI: Body mass index, HR: Heart rate.

^a^ Subjects ever diagnosed with the disorder

We hypothesized that the association between BRS and atherosclerosis is dependent on inflammation. Multiple linear regression models with 2 predictors were used to investigate the direct or indirect associations between inflammatory parameters or BRS and carotid plaque area (Table 2). The analysis showed that the standardized β-coefficient for BRS Slope, adjusted for WBCC, was attenuated by 48%, and lost its significance, whereas WBCC was attenuated by 11% and still significantly associated with carotid plaque area (Table 2). Further, the standardized β-coefficient for BRS Slope, adjusted for CRP, was attenuated by 19%, and lost its significance, whereas CRP was attenuated by 18% and still significantly associated with carotid plaque area (Table 2). This suggests that low-grade inflammation could link the association between BRS Slope and carotid plaque area.

### Independent relationships between reduced autonomic tone and inflammation, and inflammation and atherosclerosis

Given the data suggesting a relationship between autonomic tone and inflammation, and inflammation and atherosclerosis, we next investigated if these associations were independent of other clinical variables and medical therapy. Three different multivariable regression models were used to adjust for potential confounders. When adjusting for either age, or age and antihypertensive medicine, the inverse associations between WBCC and both BRS Slope and HRV SDNN, and the inverse association between CRP and BRS slope still remained significant (Table 3, Model 1–2). Further, the associations between carotid plaque area and both WBCC and CRP still remained significant (Table 3, Model 1–2). After adjusting for age, hypertensive medicine, prevalent CVD, current smoking, BMI, diabetes, hypertension, dyslipidemia, systolic blood pressure and cholesterol-lowering medicine these associations remained significant for all analysis except for the association between CRP and carotid plaque area (p = 0.093, Table 3, Model 3).

**Table 3:** Independent predictors of inflammation and atherosclerosis.

Predictor	Respons	Model 1		Model 2		Model 3	
		β	p	β	p	β	p
Log BRS Slope, ms/mm Hg	Log WBCC	-0.25	0.005	-0.25	0.006	-0.25	0.004
Log HRV SDNN, ms		-0.19	0.034	-0.18	0.047	-0.23	0.007
Log BRS Slope, ms/mm Hg	Log CRP	-0.22	0.017	-0.21	0.029	-0.23	0.013
Log HRV SDNN, ms		-0.11	0.2	-0.1	0.3	-0.1	0.3
Log CRP, mg/L	Log Plaque Area	0.2	0.024	0.18	0.038	0.14	0.093
Log WBCC, mg/L		0.3	<0.001	0.26	0.003	0.23	0.010

Model 1: Adjusted for age.

Model 2: Adjusted for age and antihypertensive medicine.

Model 3: Stepwise adjusted for prevalent CVD, current smoking, BMI, diabetes, hypertension, dyslipidemia, systolic blood pressure and cholesterol-lowering medicine. Age and antihypertensive medicine were forced into the model.

Each predictor was included in separate regression models. β-values are standardized regression coefficients. BRS: Baroreflex sensitivity, HRV: Heart rate variability, SDNN: standard deviation of RR intervals, WBCC: white blood cell count.

When separately investigating patients on cholesterol-lowering medicine and untreated patients, the interindividual associations between inflammatory parameters, carotid plaque area and parameters for autonomic function still remained for both groups, except the association between BRS Slope or HRV SDNN and WBCC, where the association was attenuated in the untreated patients (data not shown). However, the correlations did not differ significantly between treated and untreated patients.

## Discussion

This population-based study showed an inverse association between BRS and atherosclerosis and that WBCC could play an important role as a link in this relationship. Further, this study contributes with new knowledge on the independent association between autonomic function and WBCC, and between WBCC and atherosclerosis by demonstrating these relationships in the same population. Thus, this study suggests inflammation as a mediator in the relationship between reduced autonomic tone and atherosclerosis.

Autonomic dysfunction is a well-established risk factor for CVD [1–3], however the mechanisms linking autonomic tone to CVD are still unknown. Previous studies suggest a relationship between autonomic dysfunction and inflammation [4–7], as well as between inflammation and atherosclerosis [9, 10]. These findings made us hypothesize that inflammation mediates the atherogenic effects of autonomic dysfunction. Few studies have investigated the whole pathway (autonomic function—inflammation—atherosclerosis) in the same population, or carotid atherosclerosis as the primary end-point. Intima media thickness (IMT), a marker of preclinical atherosclerosis, was recently associated with increased CRP and reduced HRV in patients suffering from depression [27]. This is partly in line with the current study where we show an inverse association between autonomic function, measured by BRS, and both WBCC and established carotid plaque lesions.

The association between autonomic function and carotid plaque area could be due to direct effects of the autonomic nervous system on the progression of atherosclerosis, or mediated via other factors. To test our hypothesis that reduced autonomic function could have an impact on low-grade inflammation, which subsequently accelerates atherosclerosis, the association between BRS Slope and atherosclerosis was adjusted for the inflammatory markers WBCC and CRP. Interestingly, when adjusting for WBCC, the relationship between BRS and carotid plaque area was remarkably attenuated and no longer significant, demonstrating that WBCC could be a mechanistic link in the association between reduced BRS and carotid plaque area. However, even though adjustment for CRP also attenuates the association between reduced BRS and carotid plaque area, the role of CRP may be interpreted with care since the effect was less pronounced. Previous studies show a relationship between atherosclerosis and reduced vessel distensibility, an effect that could have an impact on autonomic function. We cannot rule out that aortic stiffness could affect autonomic function and previous studies both supports and oppose that reduced distensibility decreases BRS [28, 29]. Surprisingly, these studies rarely adjust for low-grade inflammation, known to be associated with aortic stiffness [30, 31]. Regardless of the causative direction between autonomic function and atherosclerosis, our data demonstrate that inflammation could play a role in this pathway.

Given that inflammatory markers might mediate the association between BRS and carotid plaque area, we next investigate if autonomic function was independently associated with inflammatory markers WBCC and CRP, and subsequently, if WBCC and CRP was independently associated with carotid plaque area. The hypothesis of inflammation as a mechanistic link between autonomic dysfunction and atherosclerosis derives from a number of different studies displaying an inverse association between HRV and inflammatory markers, in both patients with CVD [4–7], and in populations without CVD [32–36]. We confirm these relationships, demonstrating an association between HRV and WBCC in the current study. This relationship is also extended by including another marker of autonomic function, BRS, also demonstrating an independent association with both WBCC and CRP. In the current study, only CRP and WBCC were used to assess inflammation. Given the perplexity of the inflammatory process in atherosclerosis other inflammatory markers, such as cytokines, should be further evaluated in future studies.

The relationship between inflammatory markers and CVD has been thoroughly characterized in previous studies. WBCC, a low-grade inflammatory marker has been an independent predictor of CVD [12, 13], and associated with increased intima media thickness (IMT), as well as femoral and carotid atherosclerosis [14–17]. Our results support these findings, showing an association between WBCC and carotid plaque area, independent of other cardiovascular risk factors. Although, a large number of studies show CRP to be a strong predictor for CVD [11] this association did not remain significant after adjusting for age, hypertensive medicine and other clinical risk factors in the current study. The role of CRP as an independent predictor of the extent of atherosclerosis is debated, where some studies support this [18–20], while other studies do not [14, 37, 38]. It is possible that the relationship between CRP and CVD is more associated to the phenotype of atherosclerotic plaques [39–41], rather than the extent, which is the focus in the current study. Still, our results on inflammation and atherosclerosis confirm previous studies where carotid atherosclerosis was independently associated with WBCC but not CRP [14].

Since cholesterol-lowering treatments influence both inflammation [42, 43], and atherosclerosis [44, 45], a sensitivity analysis for cholesterol-lowering treatment was conducted. Although, most of the associations were coherent with the relationships in the whole population, the fully adjusted associations between BRS Slope or HRV SDNN and WBCC were reduced in the untreated patients (data not shown). However, the correlation in this smaller group did not differ significantly from the treated group so the lack of association is possibly due to lack of power.

In summary, in this study, men with prevalent CVD have reduced BRS, increased WBCC and more carotid atherosclerosis compared to male subjects with no history of previous CVD. We demonstrate that autonomic function is associated with atherosclerosis and that inflammation could play an important role in mediating this relationship. Although this study demonstrates that there is a relationship between autonomic dysfunction, inflammation and atherosclerosis, the causal relationship between these parameters remains to be determined.
